# Mitochondrial remodeling by extracellular vesicles co-delivering functional mitochondria and a mitophagy inducer for neuropathic pain relief

**DOI:** 10.1016/j.mtbio.2026.103636

**Published:** 2026-09-01

**Authors:** Xianquan An, Li Zhang, Xin Zhang, Meidan Fang, Cong Wang, Yu Han, Zheng Wang, Jinfeng Wang, Shuting Zuo

**Affiliations:** a Department of Anesthesiology, The Second Hospital of Jilin University, Changchun, 130041, China; b Department of Breast Surgery, The Second Hospital of Jilin University, Changchun, 130041, China; c Department of Anesthesiology and Pain Medicine, Suzhou TCM Hospital Affiliated to Nanjing University of Chinese Medicine, Suzhou, 215009, China; d CAS Key Laboratory of Nano-Bio Interface, Division of Nanobiomedicine, Suzhou Institute of Nano-Tech and Nano-Bionics, Chinese Academy of Sciences, Suzhou, 215123, China; e Jiangsu Key Laboratory of New Drug Research and Clinical Pharmacy, School of Pharmacy, Xuzhou Medical University, Xuzhou, 221004, China

**Keywords:** Neuropathic pain, Mitochondrial transplantation, Mitophagy, Extracellular vesicles, Dorsal root ganglion

## Abstract

Neuropathic pain remains a major clinical challenge due to limited efficacy and tolerability of current treatments. Mitochondrial dysfunction in dorsal root ganglion (DRG) cells is recognized as a key pathogenic mechanism, but effective strategies to restore mitochondrial homeostasis are lacking. Here, we first identified profound deficits in mitochondrial quantity and quality in DRG neurons and satellite glial cells (SGCs) from a chemotherapy-induced peripheral neuropathy (CIPN) model. To address this, we developed an extracellular vesicle-based nanoplatform (EVs@Mi/UR) loaded with a mitophagy inducer, which integrates exogenous mitochondrial transplantation with mitophagy induction. EVs@Mi/UR not only increased mitochondrial mass in DRG neurons and SGCs through efficient mitochondrial transplantation, but also improved mitochondrial quality by eliminating damaged organelles, thereby enhancing mitochondrial respiration and metabolic function. In both CIPN and spared nerve injury (SNI) mouse models, EVs@Mi/UR significantly alleviated mechanical allodynia, thermal hyperalgesia, and cold hypersensitivity with superior efficacy. Notably, even in SNI models that did not exhibit baseline mitochondrial deficits, EVs@Mi/UR still produced analgesic effects by improving mitochondrial quality. This work establishes mitochondrial remodeling as a promising strategy for neuropathic pain and provides a translatable EV-based nanoplatform for dual-modality mitochondrial intervention.

## Highlights

• A mitochondria-contained EV was developed to preload mitophagy inducers for achieving exogenous mitochondrial transplantation and mitophagy induction in DRG neurons and SGCs.
• The EVs-based nanoplatform restores the mitochondrial homeostasis in DRG neurons and SGCs of CIPN mice through transferring exogenous mitochondria and eliminating damaged mitochondria, thereby alleviating the CIPN-induced neuropathic pain.
• The nanoplatform exerts analgesic effects even in SNI models without baseline mitochondrial deficits by improving mitochondrial quantity and quality, demonstrating the mitochondrial remodeling as a promising strategy for neuropathic pain.

## Graphical abstract

**Figure:**
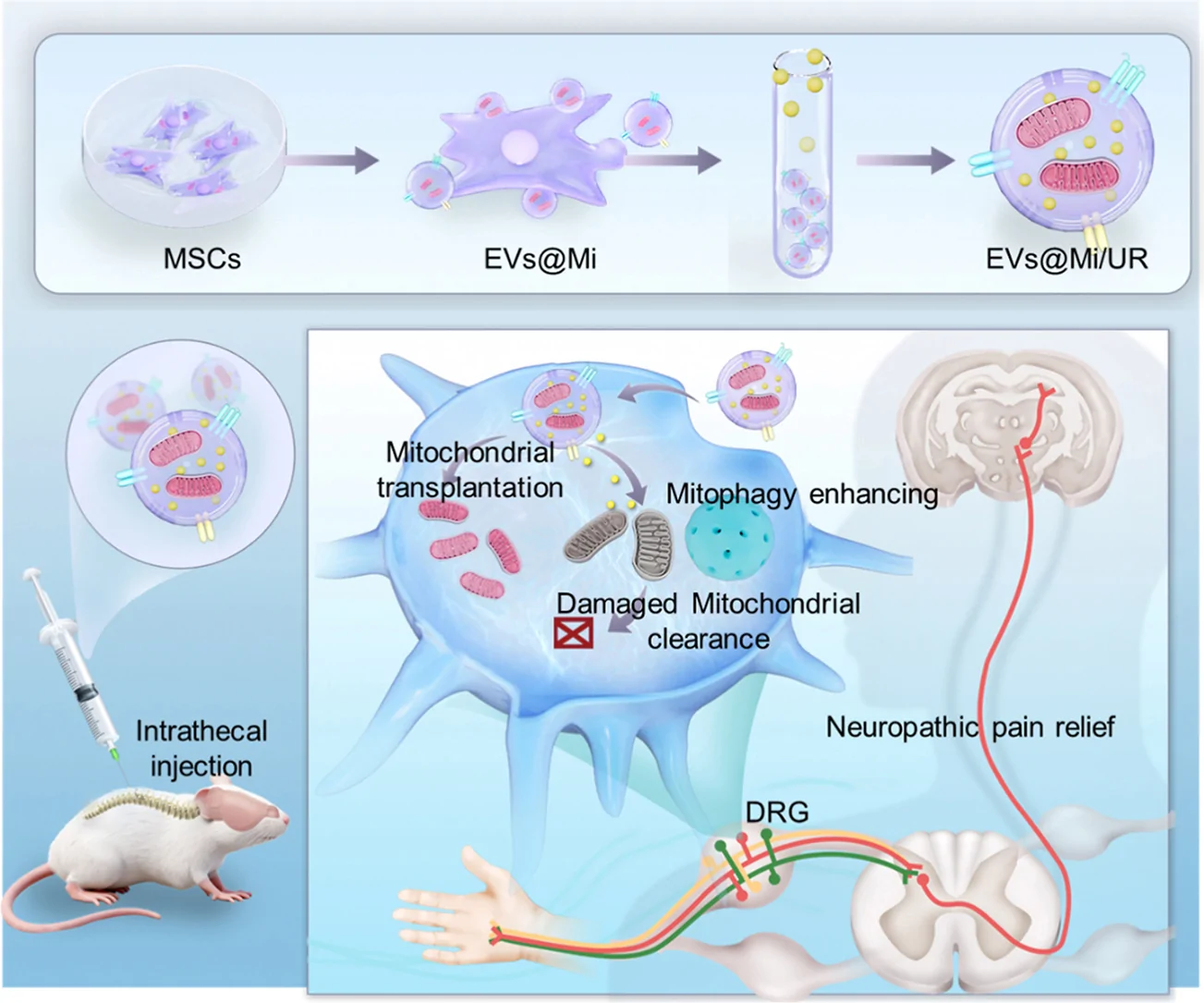

## 1 Introduction

**Scheme 1:**
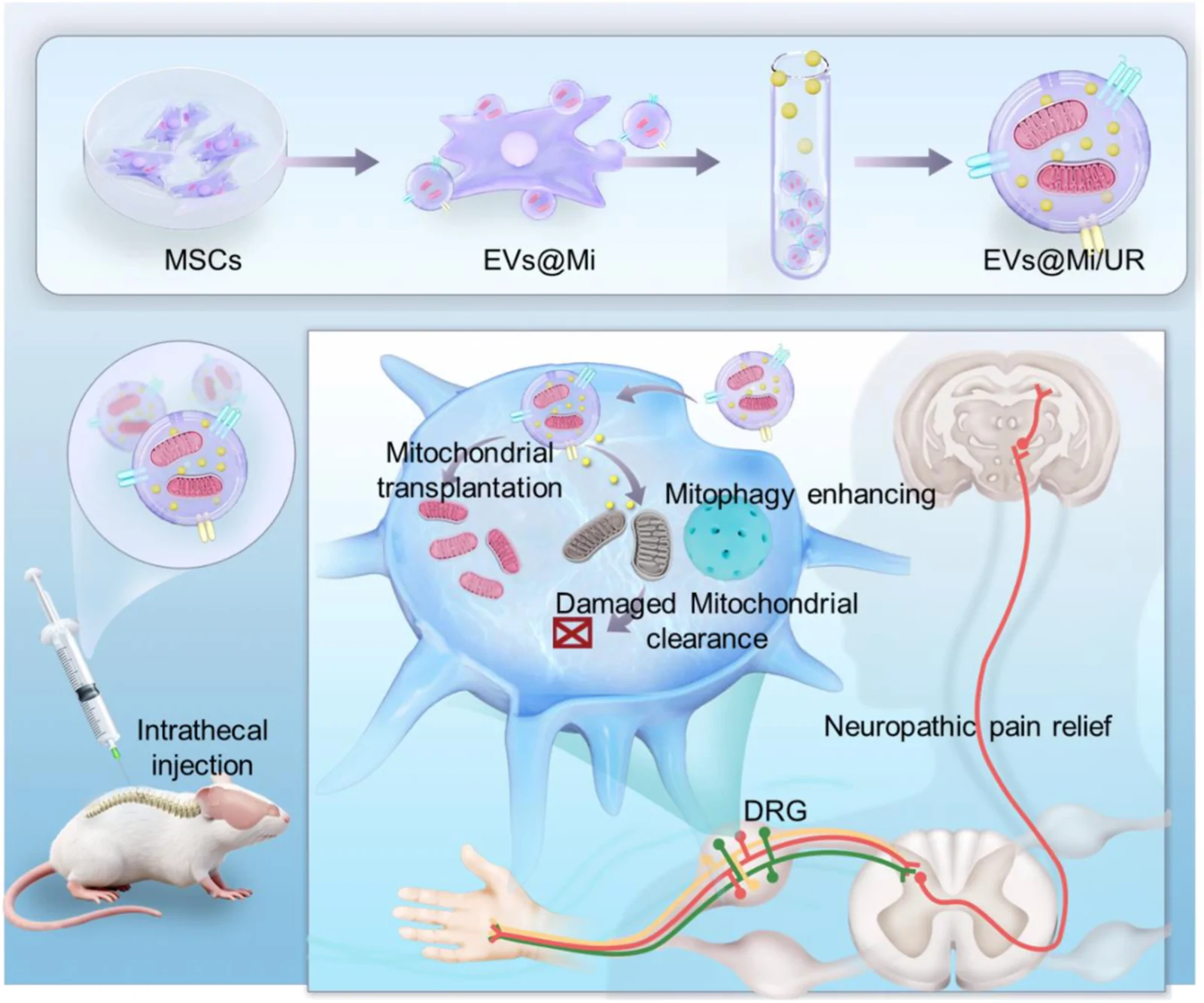
Construction of mitochondria-contained EVs with Urolithin A loading, to retore the mitochondrial homeostasis balance of DRG cells for the relief of neuropathic pain through simultaneous achieving exogenous mitochondrial supplement and endogenous mitochondria renewal.

Neuropathic pain arises from a lesion or disorder of the somatosensory system and manifests as spontaneous ongoing or lancinating pain, which can be triggered or intensified by noxious as well as non-noxious stimuli [[1], [2], [3]]. Due to global population aging, rising diabetes prevalence, and improved post-treatment cancer survival, the incidence of neuropathic pain continues to rise [4,5]. At present, this condition represents the most refractory form of chronic pain, affecting approximately 3–17% of people worldwide; nonetheless, available therapeutic options exhibit limited effectiveness and suboptimal tolerance [[6], [7], [8]]. Current management primarily relies on pharmacologic agents: anticonvulsants and antidepressants for mild pain, and local anesthetics or opioid analgesics for moderate to severe cases. Yet, fewer than 50% of patients respond to these treatments, and among responders, only weak-to-moderate pain relief is typically achieved [6,[9], [10], [11], [12], [13]]. Dose-related adverse effects and the development of tolerance often prevent dose escalation needed for sustained analgesic efficacy. Hence, developing effective non-pharmacological alternatives for neuropathic pain management is an urgent priority (see Scheme 1).

Neuropathic pain can originate in the peripheral nervous system (PNS), specifically within the dorsal root ganglia (DRG) that contain the cell bodies of primary sensory neurons known as nociceptors [[14], [15], [16], [17], [18], [19]]. These neurons with long peripheral branches innervating target organs such as the skin, detect and relay somatosensory information, such as to the central nervous system. Owing to their essential role in pain signaling, distinctive anatomical location, and amenability to targeted therapies, primary sensory neurons in DRGs represent attractive targets for chronic pain treatment [[20], [21], [22]]. Mitochondria serve as the central metabolic hub supporting cellular energy production and metabolic fitness. Sensory neurons have exceptionally high metabolic demands and are particularly susceptible to mitochondrial dysfunction under neuropathic pain conditions [[23], [24], [25], [26], [27]]. Consistent with this vulnerability, disrupted mitochondrial homeostasis has emerged as a common feature of peripheral neuropathies, such as chemotherapy-induced peripheral neuropathy (CIPN), which is characterized by axonal degeneration, sensory loss, and chronic pain. Therefore, strategies aimed at restoring mitochondrial homeostasis and enhancing mitochondrial function are highly sought for neuropathic pain management. Mitochondrial transplantation, which involves the transfer of exogenously derived healthy mitochondria into damaged cells, has been proposed as a feasible approach to rescue mitochondrial dysfunction in DRG cells [26,[28], [29], [30], [31]]. Despite its promise, substantial barriers to in vivo mitochondrial transplantation remain, including loss of mitochondrial activity, poor targeting, limited uptake by DRG cells, and recognition of the foreign organelles. Moreover, simply boosting mitochondrial quantity via exogenous transfer is insufficient; alongside augmenting functional mitochondria, eliminating dysfunctional ones is equally critical for restoring mitochondrial homeostasis in DRG cells [[32], [33], [34], [35]]. This elimination process relies on mitophagy, a selective autophagic pathway that degrades damaged or fragmented mitochondria and thereby supports cellular homeostasis [32,36]. Consequently, a combined strategy that integrates mitochondrial transplantation with mitophagy induction may be more effective in restoring mitochondrial balance and alleviating neuropathic pain.

Extracellular vesicles (EVs) function as mediators of mitochondrial transport across cells, making them ideal natural vectors for mitochondrial delivery [37]. Compared to naked mitochondria, EV-encapsulated mitochondria benefit from the protective lipid bilayer, which enhances their resistance to Ca^2+^ overload and oxidative stress. In this work, we identified that the DRG sensory neurons and satellite glial cells (SGCs) in CIPN models had poor mitochondrial quantity and qualities relative to normal mice. Then, we developed mitochondria-contained EVs, further encapsulating the Urolithin A, a mitophagy inducer, to simultaneous achieve exogenous mitochondrial supplement and endogenous mitochondria renewal. The nanoplatform (EVs@Mi/Ur) not only increased the mitochondrial content, but also decreased damaged mitochondria in DRG sensory neurons and SGCs, thus improving mitochondrial status and metabolic capability. The in vivo results demonstrated that intervention with the nanoplatform that integrating mitochondrial transplantation and mitophagy induction, led to superior analgesic effects in CIPN models, compared with mitochondrial transplantation alone. Furthermore, we found that in the spared nerve injury (SNI) model, DRG cells did not exhibit the mitochondrial dysfunction observed in the CIPN model. Nevertheless, our nanoplatform alleviated SNI-induced pain through increasing mitochondrial number and quality in DRG cells. Collectively, our work establishes mitochondrial remodeling as a promising strategy for the relief of neuropathic pain, and provided an effective nanoplatform for mitochondrial transplantation in DRG cells.

## 2 Methods

### 2.1 Mouse DRG neuron and SGCs culture

Primary DRG from mice were dissociated enzymatically using collagenase (0.2 mg/mL, Sigma, 10103578001) and Dispase-II (3 mg/mL, Sigma, D4693) for 60 min at 37 °C. After trituration with a pipette, the cell suspension was passed through a 70-μm nylon mesh and centrifuged at 300×g for 5 min. Neurons were seeded onto glass coverslips pre-coated with 0.1 mg/mL poly-D-lysine (Thermo, A3890401) and cultured in Neurobasal medium containing 10% FBS, 2% B27 supplement, and 1% penicillin-streptomycin (termed Neurobasal-supplemented medium) at 37 °C in 5% CO_2_. To purify SGCs, DRG were digested with the same enzymes for 90 min at 37 °C, then mechanically dissociated, filtered, and centrifuged as above. Cells were plated on uncoated 35-mm dishes (VWR, 10861-656) and maintained in Neurobasal-supplemented medium. On day 3 in vitro (DIV3), non-SGCs (including neurons) were removed by vigorous hand-shaking of the dishes for 15-20 s, leaving an adherent, purified SGC layer. DRG sensory neurons were seeded at 1.5 × 10^4^ cells/cm^2^ on poly-D-lysine-coated coverslips or plates, while SGCs were seeded at 1.5 × 10^4^ cells/cm^2^ on uncoated dishes. These densities were consistently applied across all downstream functional assays, including MTDR, MitoSOX, TMRM, JC-1, and Seahorse OCR measurements.

### 2.2 CIPN-induced neuropathic pain model

Male C57BL/6 J mice (6-8 weeks) were purchased from Nanjing Sikerui Biological Technology Co., Ltd. All the animal experiments were approved by the Animal Ethics Committee of the Chinese Academy of Sciences. Paclitaxel (Ptx) powder was first dissolved in sterile PBS (pH 7.4) containing 4% DMSO and 4% Tween-80 to obtain a 1 mg/mL stock. C57BL/6 J mice received intraperitoneal injections of this stock at 2 mg Ptx per kg body weight every other day, for a total cumulative dose of 8 mg/kg over 6 days.

### 2.3 SNI-induced neuropathic pain model

Peripheral neuropathic pain was induced by spared nerve injury (SNI) surgery. Under 3% isoflurane inhalation anesthesia, the hind limb of a C57 mouse was incised to expose the three branches (common peroneal, tibial, and sural nerves) of the sciatic nerve. The common peroneal and tibial nerves were tightly ligated with 6-0 silk, while the sural nerve was left intact. The wound was closed, and mice were allowed to recover. Sham-operated animals underwent the same procedure without nerve ligation.

### 2.4 Preparation of EVs@Mi/UR (or EVs@Mi/UR)

MSCs used for EV production were isolated from the adipose tissues of C57BL/6J mice. Briefly, adipose tissues were minced and digested with 0.075% collagenase type I in DMEM at 37 °C for 120 min with intermittent shaking. The digestion was neutralized by adding an equal volume of DMEM containing 10% FBS, and the mixture was centrifuged at 1500 rpm for 10 min. The pellet was resuspended, filtered through a 100-μm cell strainer, and plated in DMEM supplemented with 10% FBS and 1% penicillin-streptomycin. After 72 h, non-adherent cells were removed, and adherent cells were cultured until 80% confluence. Cells, exhibiting typical fibroblast-like morphology and positive for CD29/CD44/Sca-1 (negative for CD34/CD45), were used for EV production. MSCs at passage 3 (approximately 60% confluence) were washed twice with PBS and then cultured in medium containing 10% exosome-depleted fetal bovine serum. After 48 h, the conditioned medium was collected and centrifuged at 300×g for 5 min to remove dead cells. The supernatant was further centrifuged at 2000×g for 10 min to eliminate apoptotic bodies and debris, followed by two rounds of centrifugation at 20,000×g for 60 min. The resulting pellet (EVs@Mi) was resuspended in PBS. All steps were performed at 4 °C to preserve mitochondrial activity. Urolithin A (UA) was loaded into EVs@Mi to produce EVs@Mi/UR. (Note: the preparation of UA-loaded formulation followed the same ultracentrifugation steps, with UA added during the final resuspension.)

### 2.5 Characterization of EVs@Mi/UR

Size distribution and concentration were determined by nanoparticle tracking analysis (NTA) using a ZetaView Particle Tracker (Particle Metrix, Germany). Morphology was examined by transmission electron microscopy (TEM, HT7800; Hitachi). Surface marker CD9 and mitochondrial outer membrane protein Tom20 were analyzed by capillary immunoassay (ProteinSimple; Biotechne, USA).

### 2.6 Cytotoxicity assessment

DRG sensory neurons and SGCs were seeded in 96-well plates at 5 × 10^3^ cells/well and incubated with escalating concentrations of EVs@Mi or EVs@Mi/UR for 24 h. Cell viability was measured by MTT assay (3-(4,5-dimethylthiazol-2-yl) −2,5-diphenyltetrazolium bromide, Sigma).

### 2.7 Cellular uptake assay

For confocal imaging, cells were seeded in 6-well plates (5 × 10^5^/well) and incubated with 50 μg/mL of MTG-labeled EVs@Mi, EVs@Mi/UR, or free mitochondria for 3 h. After washing with PBS, cells were fixed with 4% paraformaldehyde for 15 min, stained with DAPI (1 μg/mL) for 10 min, and imaged with a confocal laser scanning microscope (Zeiss LSM 880). For flow cytometric quantification, treated cells were harvested, washed, resuspended in FACS buffer, and analyzed on a Beckman Coulter cytometer.

### 2.8 Measurement of mitochondrial mass (MTDR staining)

Mitochondrial mass in DRG neurons and SGCs was assessed using MitoTracker Deep Red (MTDR, Thermo Fisher). Cells were incubated with 50 nM MTDR in culture medium for 30 min at 37 °C, washed twice with PBS, and then analyzed either by flow cytometry (for mean fluorescence intensity, MFI) or by confocal microscopy. For in vivo samples, dissociated DRG cells were stained with MTDR following the same protocol.

### 2.9 Detection of mitochondrial reactive oxygen species (mitoROS)

MitoSOX Red (Thermo Fisher) was used to evaluate mitochondrial superoxide levels. Cells were incubated with 5 μM MitoSOX in HBSS for 10 min at 37 °C, protected from light. After two washes with PBS, the fluorescence was measured by flow cytometry (MFI) or visualized under a confocal microscope.

### 2.10 Assessment of mitochondrial membrane potential

For TMRM staining, cells were loaded with 100 nM tetramethylrhodamine methyl ester perchlorate (TMRM, Thermo Fisher) in HBSS for 30 min at 37 °C, then washed and immediately analyzed by flow cytometry. JC-1 assay was performed using a kit (Beyotime Biotechnology). After washing cells with PBS, 1 mL of JC-1 working solution was added and mixed. Following 20 min incubation at 37 °C, the supernatant was removed, cells were washed twice with JC-1 staining buffer, and then imaged by confocal microscopy or quantified by flow cytometry (ratio of red J-aggregate to green monomer).

### 2.11 Quantification of mtDNA copy number

Total DNA was extracted from isolated DRG sensory neurons or SGCs using a DNeasy Blood & Tissue Kit (Qiagen). Real-time qPCR was performed with primers targeting mitochondrial-encoded ND1 and nuclear-encoded B2m (as reference). Relative mtDNA copy number was calculated as 2^(ΔCt), where ΔCt = Ct(ND1) – Ct(B2m).

### 2.12 Quantitative real-time PCR (qPCR) for gene expression

Total RNA was extracted using TRIzol reagent (Invitrogen) and reverse transcribed into cDNA with a PrimeScript RT kit (Takara). qPCR was run on a LightCycler 480 system using SYBR Green Master Mix (Roche). Primers for Pgc-1α, Pgc-1β, Tfam, Pink1, Prkn, and housekeeping gene Gapdh were designed as previously described. Relative expression was calculated by the 2^(-ΔΔCt) method.

### 2.13 Oxygen consumption rate (OCR) measurement

OCR was measured with an XFe96 Seahorse Extracellular Flux Analyzer (Agilent). Primary DRG neurons were seeded in XFe96 cell culture microplates (Agilent, 101085-004) and incubated at 37 °C with 5% CO_2_. On the assay day, cells were washed and placed in Seahorse XF base medium (Agilent, 103575-100) supplemented with 10 mM glucose, 1 mM pyruvate, and 2 mM glutamine. Each measurement cycle consisted of 3 min mixing, 1 min waiting, and 3 min recording. After three baseline measurements, oligomycin (0.5 μM), FCCP (2 μM), and rotenone/antimycin A (0.5 μM each) were sequentially injected. OCR values were normalized to cell number. For fresh tissue, isolated DRG were placed in XFe96 spheroid microplates containing the same base medium; oligomycin (12 μM), FCCP (20 μM), and rotenone/antimycin A (20 μM each) were injected, and OCR was normalized to protein content.

### 2.14 In vivo tracing of DiR-labeled EVs@Mi/UR

EVs@Mi and EVs@Mi/UR were labeled with DiR (MedChemExpress, HY-D1048) and then pelleted by ultracentrifugation at 120,000×g for 120 min. After washing with PBS, the labeled vesicles were resuspended and administered intrathecally into mice. Whole-body fluorescence imaging was performed using an IVIS system (PerkinElmer) on days 0, 2, 4, 6, and 8 post-injection. Maximum radiant efficiency and maximal diffusion distance from the injection site (measured on day 2) were quantified using IVIS software.

### 2.15 Behavioral tests

#### 2.15.1 Von-Frey test (mechanical allodynia)

Mice were habituated on a metal mesh shelf for 1 h daily over 5 days. On test day, each mouse was placed in a plexiglas chamber (8 × 8 × 8 cm) for 30 min, then von-Frey filaments (0.008-1 g) were applied to the plantar surface of the right hind paw. The paw withdrawal threshold (PWT) was determined by the up-down method.

#### 2.15.2 Hargreaves test (thermal hyperalgesia)

Mice were acclimated for 30 min on an elevated transparent glass platform. A radiant heat source (Ugo Basile, #37370) was directed at the plantar surface through the glass. The heat was automatically turned off upon paw withdrawal or after 20 s to avoid tissue damage. Each paw was tested three times with a 10-min interval, and the mean withdrawal latency was recorded.

#### 2.15.3 Cold pain test

A dry-ice assay was used. Mice were placed on a 5-mm glass platform inside a test box. Small pieces of dry ice were loaded into a 5 mL syringe and positioned under the glass directly beneath the hind paw. The latency for paw withdrawal from the cold stimulus was measured. Each hind paw was tested three times, and the average was used for analysis.

### 2.16 Statistical analysis

All data are presented as mean ± SD. Comparisons between two groups were performed using unpaired two-tailed Student's t-test; multiple groups were compared by one-way or two-way ANOVA followed by Tukey's post-hoc test. A p-value < 0.05 was considered statistically significant.

## 3 Results

**Fig. 1:**
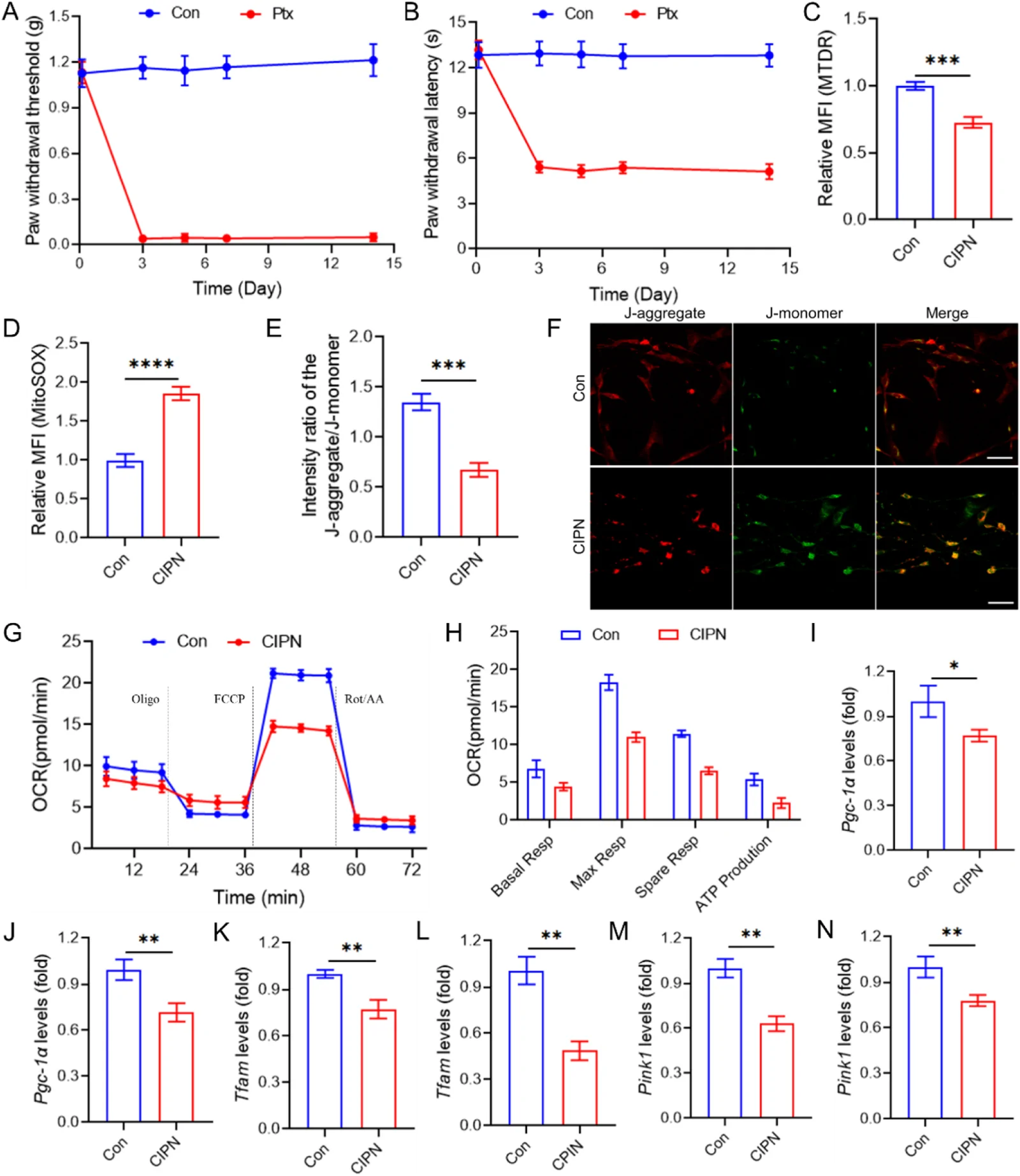
**Mitochondrial status of DRG cells in CIPN models.** (A) Mechanical allodynia examination of mice treated with Ptx, n = 5. (B) Thermal hyperalgesia test of mice treated with Ptx, n = 5. (C) Relative mean fluorescence intensity (MFI) of MTDR in DRG sensory neurons of CIPN model mice, n = 3. (D) Relative MFI of MitoSOX Red in DRG sensory neurons of CIPN model mice, n = 3. (E) Flow cytometry analysis of the intensity ratio of the J-aggregate/J-monomer in DRG sensory neurons of healthy and CIPN mice, n = 3. (F) Confocal images of mitochondrial membrane potential in DRG sensory neurons of healthy and CIPN mice, the scale bar = 20 μm. (G) Oxygen consumption rates (OCR) of DRG sensory neurons after treated with various mitochondrial inhibitors, n = 3. (H) Quantitative analysis of basal, maximal, and spare respiratory capacity and ATP production rate of DRG sensory neurons according to the OCR, n = 3. (J-N) Quantitative qPCR analysis of mitochondrial biogenesis genes including *Pgc-1α* (I, J), *Tfam* (K, L), and *Pink1* (M, N) in the DRG sensory neurons (I, K and M) and SGCs (J, L and N). Data are expressed as mean ± SD, *p < 0.05, **P < 0.01, ***p < 0.001, ****P < 0.0001.

Given that neural activity depends on precisely regulated mitochondrial function, we began our research by exploring mitochondrial status in DRG from CIPN mice. Mechanical allodynia and thermal hyperalgesia tests showed that repeated intraperitoneal paclitaxel (Ptx) injections led to pronounced reductions in paw withdrawal threshold and latency, confirming successful CIPN induction (Fig. 1A and B). Then, we isolated sensory neurons and satellite glial cells (SGCs) from DRG of CIPN models and detected the mitochondrial mass of these using MitoTracker Deep Red (MTDR). Both sensory neurons and SGCs from CIPN mice exhibited lower MTDR signals than those from healthy controls (Fig. 1C and S1). Consistently, mtDNA copy numbers were also lower in those from CIPN models (Fig. S2). Furthermore, MitoSOX staining revealed elevated mitochondrial reactive oxygen species (mitoROS) levels in SGCs and neurons from CIPN mice, suggesting the increased oxidative stress and mitochondrial injury in DRG of CIPN (Fig. 1D and S3). JC-1 probe analysis showed a marked reduction in the J-aggregate/J-monomer fluorescence ratio of sensory neurons and SGCs isolated from CIPN mice (Fig. 1E–F and S4). Additionally, tetramethylrhodamine methyl ester perchlorate (TMRM) indicated sensory neurons and SGCs from the CIPN mice had lower mitochondrial membrane potential compared with those from normal mice (Fig. S5), indicating the accumulation of dysfunctional mitochondria in DRG of CIPN mice. These results suggest that CIPN impairs both the quantity and quality of mitochondria within DRGs. Consistent with these alterations, mitochondrial metabolic function was severely compromised in DRG sensory neurons from CIPN mice (Fig. 1G). Measurements of cellular oxygen consumption revealed significant declines in basal, maximal, spare respiratory capacities, ATP production as well as coupling efficiency, indicating broadly diminished mitochondrial bioenergetics in DRG neurons of CIPN animals (Fig. 1H and S6). Quantitative PCR also showed reduced levels of biogenesis-related genes including *Pgc-1α*, *Pgc-1β*, and *Tfam*, as well as mitophagy-related genes, such as *Pink1* and *Prkn*, indicating the impaired mitochondrial regeneration and renewal in DRG sensory neurons of CIPN mice (Fig. 1I–N and S7). Collectively, these data demonstrate severe deficits in mitochondrial mass, function, and regeneration in DRGs of CIPN mice.

**Fig. 2:**
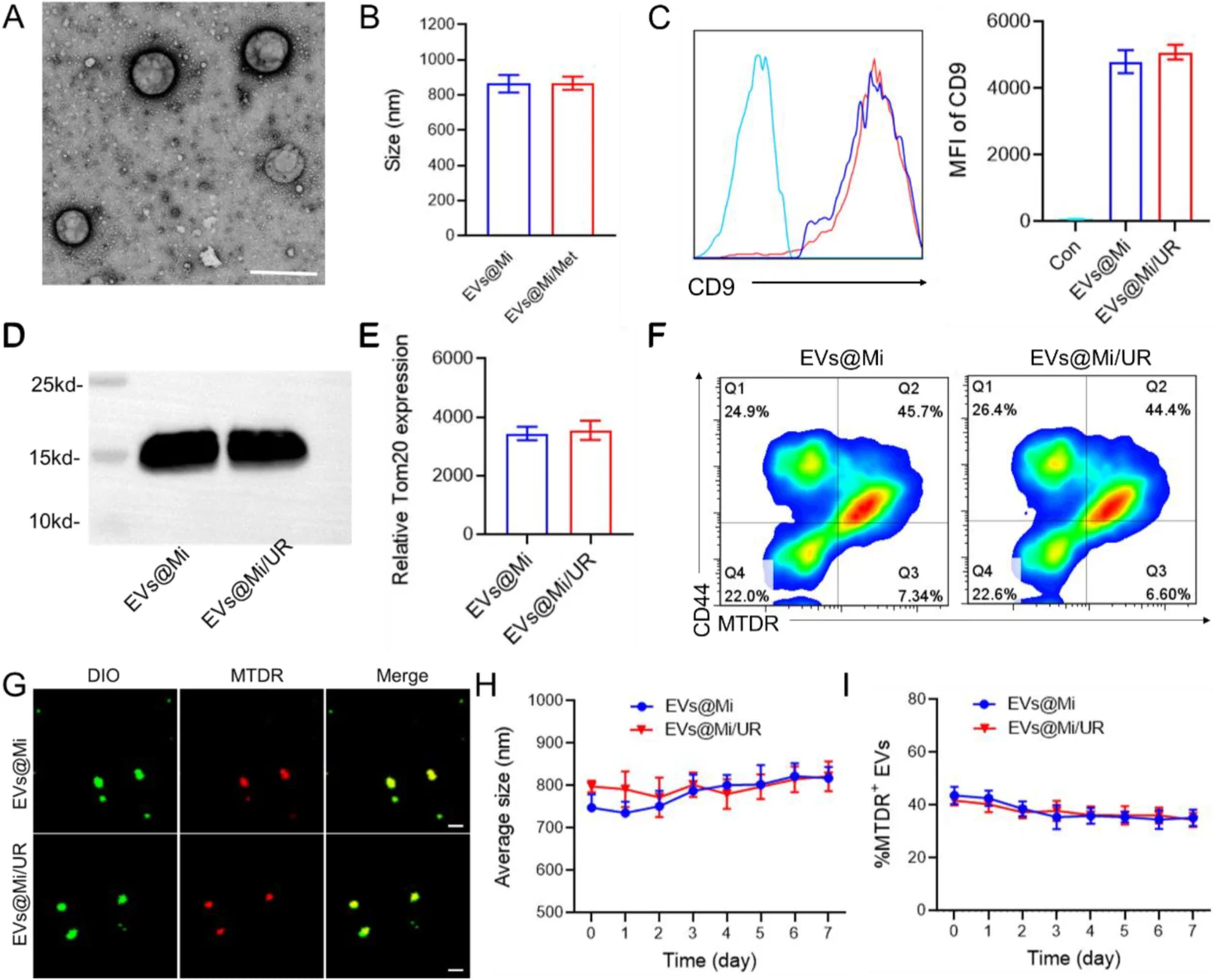
**Preparation of EVs@Mi/UR.** (A) TEM images of EVs@Mi, the scale bar = 1 μi. (B) The size distribution of EVs@Mi and EVs@Mi/UR, n = 3. (C) Flow cytometry analysis of the expression of CD9 on the surface of EVs@Mi and EVs@Mi/UR, n = 3. (D) Tom20 expression levels in EVs@Mi and EVs@Mi/UR. (E) Quantitative analysis of Tom20 expression by capillary immunoassays, n = 3. (F) Flow cytometry analysis of CD44+MTDR + EVs in EVs@Mi and EVs@Mi/UR. (G) Colocalization of MTDR-stained mitochondria with DIO labeled EVs, the scale bar = 1 μ 1 (H) Long-term stability of EVs@Mi and EVs@Mi/UR after 7 days of stockage in PBS, n = 3. (I) Mitochondrial activity of EVs@Mi and EVs@Mi/UR for 7 days, n = 3. Data are expressed as mean BS, n = 3.

Having identified the poor mitochondrial status in sensory neurons, we proposed a combinatorial strategy that integrated exogenous mitochondrial transfer with endogenous damaged mitochondrial clearance to restore DRG mitochondrial homeostasis for alleviating CIPN. MSCs are readily accessible, derive from multiple sources, and harbor highly active mitochondria, making them ideal for obtaining mitochondria-containing extracellular vesicles (EVs). We isolated such vesicles (EVs@Mi) from MSCs via differential centrifugation. Transmission electron microscopy (TEM) and nanoparticle tracking analysis (NTA) showed typical cup-shaped morphology with a size of approximately 850 nm, which falls within the size range of microvesicles (100-1000 nm) rather than small exosomes (Fig. 2A and B). The expression of the EV markers CD9 on the EVs@Mi was verified by flow cytometry (Fig. 2C) and the mitochondrial membrane protein Tom20 was detected in EVs@Mi (Fig. 2D and E). Furthermore, active mitochondria that were labeled with MTDR, were observed to localize within the DIO-labeled EVs (Fig. 2G). These results confirmed the presence of active mitochondria in the EVs@Mi. We further investigated the percentage of EVs@Mi containing active mitochondria using flow cytometry. The proportion of mitochondria-positive EVs (CD44^+^MTDR^+^) reached 45.7% (Fig. 2F). Then, we loaded Urolithin A (UR), a well-recognized mitophagy inducer, into EVs@Mi to prepare EVs@Mi/UR. This formulation exhibited high drug loading efficiency (9.81%) and the encapsulation of UR didn't affect the size and mitochondria activity within EVs@Mi/UR (Fig. 2C–G). Additionally, both EVs@Mi and EVs@Mi/UR remained stable without aggregation after 7 days in PBS, and encapsulated mitochondria retained activity during this period, indicating favorable colloidal stability and bioactivity for medical use (Fig. 2H and I).

**Fig. 3:**
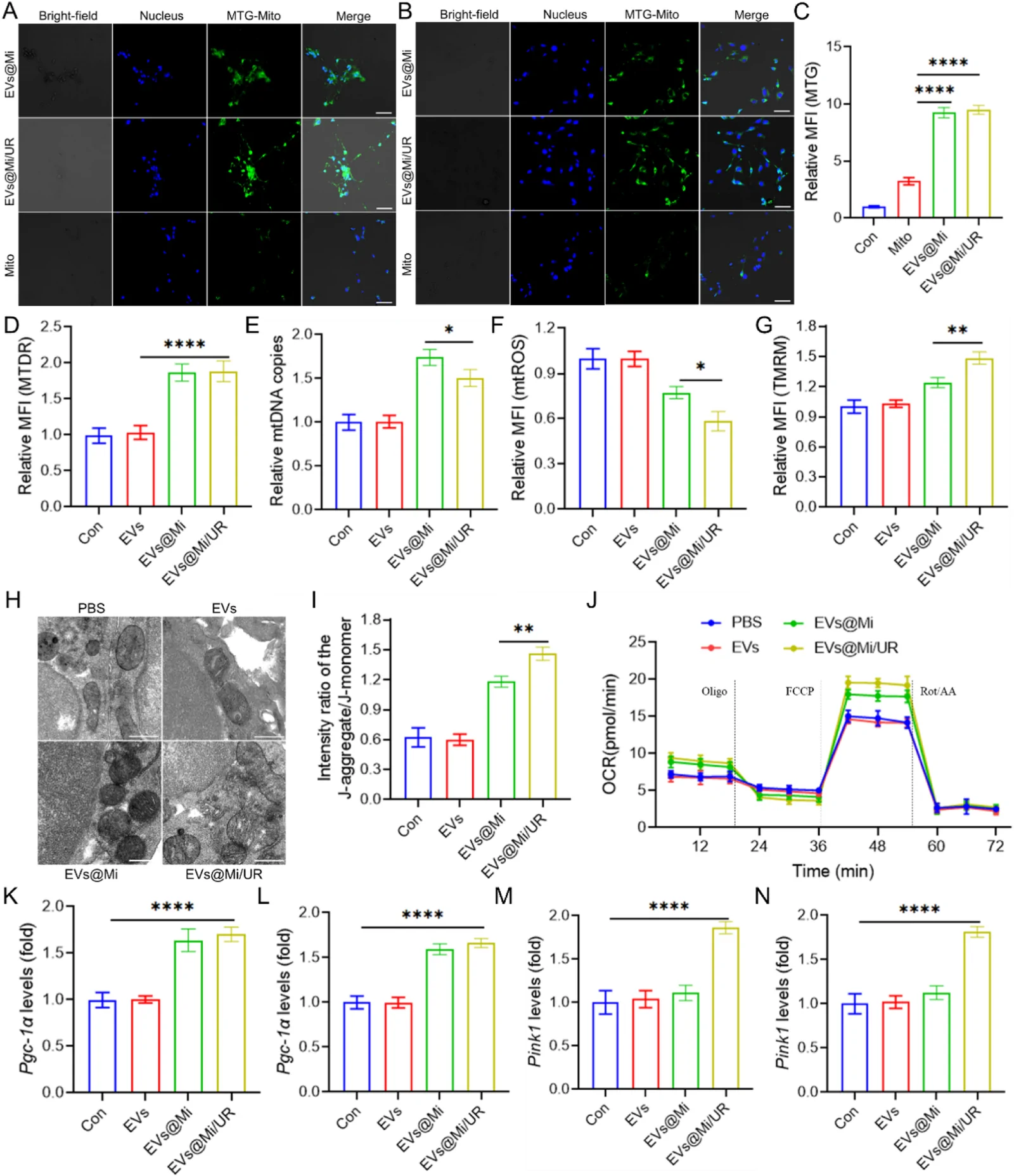
(A) Cellular internalization of MTG-labeled EVs@Mi, EVs@Mi/UR and free mitochondria after incubation with sensory neurons (A) and SGCs (B) for 3 h; scale bar = 20 μm. (C) Quantitative analysis of the cellular internalizaqtion of EVs@Mi, EVs@Mi/UR and free mitochondria with DRG sensory neurons after 3 h of incubation, n = 3. (D) Relative MFI of MTDR in DRG sensory neurons after various treatments, n = 3. (E) Relative counts of mtDNA copies in sensory neurons, n = 3. (F) Relative MFI of MitoSOX Red in DRG sensory neurons after various treatments. (G) Relative MFI of TMRM in sensory neurons after various treatments, n = 3. (H) TEM images of mitochondrial morphology in sensory neurons after various treatments, the scale bar = 500 nm. (I) Flow cytometry analysis of the intensity ratio of the J-aggregate/J-monomer in DRG sensory neurons after various treatments, n = 3. (J) OCR of DRG sensory neurons subjected to various mitochondrial inhibitors after various treatments, n = 3. (K-N) Quantitative qPCR analysis of mitochondrial biogenesis genes including *Pgc1α* (K, L) and *Pink1* (M, N) in the DRG sensory neurons (K and M) and SGCs (L and N). Data are expressed as mean Data are expressed as mean treatments, n = 3. (K-N).

The biocompatibility of EVs@Mi and EVs@Mi/UR was evaluated in isolated DRG sensory neurons and SGCs. Cell viability exceeded 90% for both EVs@Mi and EVs@Mi/UR even at the concentration of 80 μg/mL, indicating their low cytotoxicity (Fig. S8). Subsequently, we detected the cellular internalization of EVs@Mi in DRG sensory neurons and SGCs. Confocal microscopy and flow cytometry showed efficient uptake of EVs@Mi and EVs@Mi/UR by SGCs and sensory neurons (Fig. 3A–C and S9). Additionally, EVs@Mi and EVs@Mi/UR exhibited higher internalization efficiency than free mitochondria, underscoring the delivery advantage of MSC-derived vesicles for mitochondrial transplantation. To assess mitochondrial remodeling capacity, we added a control group using MSC vesicles without mitochondria (EVs). Compared with EVs, both EVs@Mi and EVs@Mi/UR significantly elevated mitochondrial mass in sensory neurons and SGCs isolated from CIPN mice (Fig. 3D and S10A). EVs@Mi/UR exhibited a comparable MTDR intensity but a slightly lower mtDNA copies relative to EVs@Mi, (Fig. 3E and S10B). It could be explained that MTDR signals depend on an intact mitochondrial membrane potential and therefore labels functional mitochondria, whereas mtDNA qPCR quantifies total mitochondrial genomes, which include those residing in damaged or depolarized organelles. The UR in the EVs@Mi/UR selectively triggers the clearance of dysfunctional mitochondria with compromised mtDNA, leading to a reduction in the total mtDNA pool, yet maintains the functional mitochondrial mass at a level similar to that of EVs@Mi. As expected, EVs@Mi/UR elevated the TMRM signals and JC-1 red/green ratio, and reduced mitoROS levels in sensory neurons and SGCs, which was more pronounced than EVs@Mi, further indicating that EVs@Mi/UR decreased the damaged mitochondria in sensory neurons and SGCs (Fig. 3F–G, 3I and S11). Furthermore, EVs@Mi/UR improved the density of mitochondrial cristae compared with EVs and PBS, evidenced by increased folds/wrinkles, which was more efficient than EVs@Mi (Fig. 3H). Additionally, both EVs@Mi/UR and EVs@Mi upregulated the levels of mitochondrial biogenesis-related genes, possibly owing to introduction of exogenous mitochondrial (Fig. 3K–L and S12). However, EVs@Mi exerted weak influence on mitophagy. Levels of key mitophagy-related gene remained relatively low levels. In contrast, EVs@Mi/UR significantly upregulated the levels of *Pink1* in DRG cells, further indicated that EVs@Mi/UR could enhance mitophagy for prompting the clearance of damaged mitochondria (Fig. 3K–N). OCR analysis confirmed that EVs@Mi/UR and EVs@Mi improved basal, maximal, and spare respiration, ATP production as well as coupling efficiency in DRG sensory neurons from CIPN models, with EVs@Mi/UR showing superior effects relative to EVs@Mi (Fig. 3J and S13-14). Collectively, these results indicate that EVs@Mi/UR not only boosts mitochondrial quantity and quality, but also enhances mitochondrial functions through exogenous mitochondrial transplantation and endogenous mitochondrial regeneration.

**Fig. 4:**
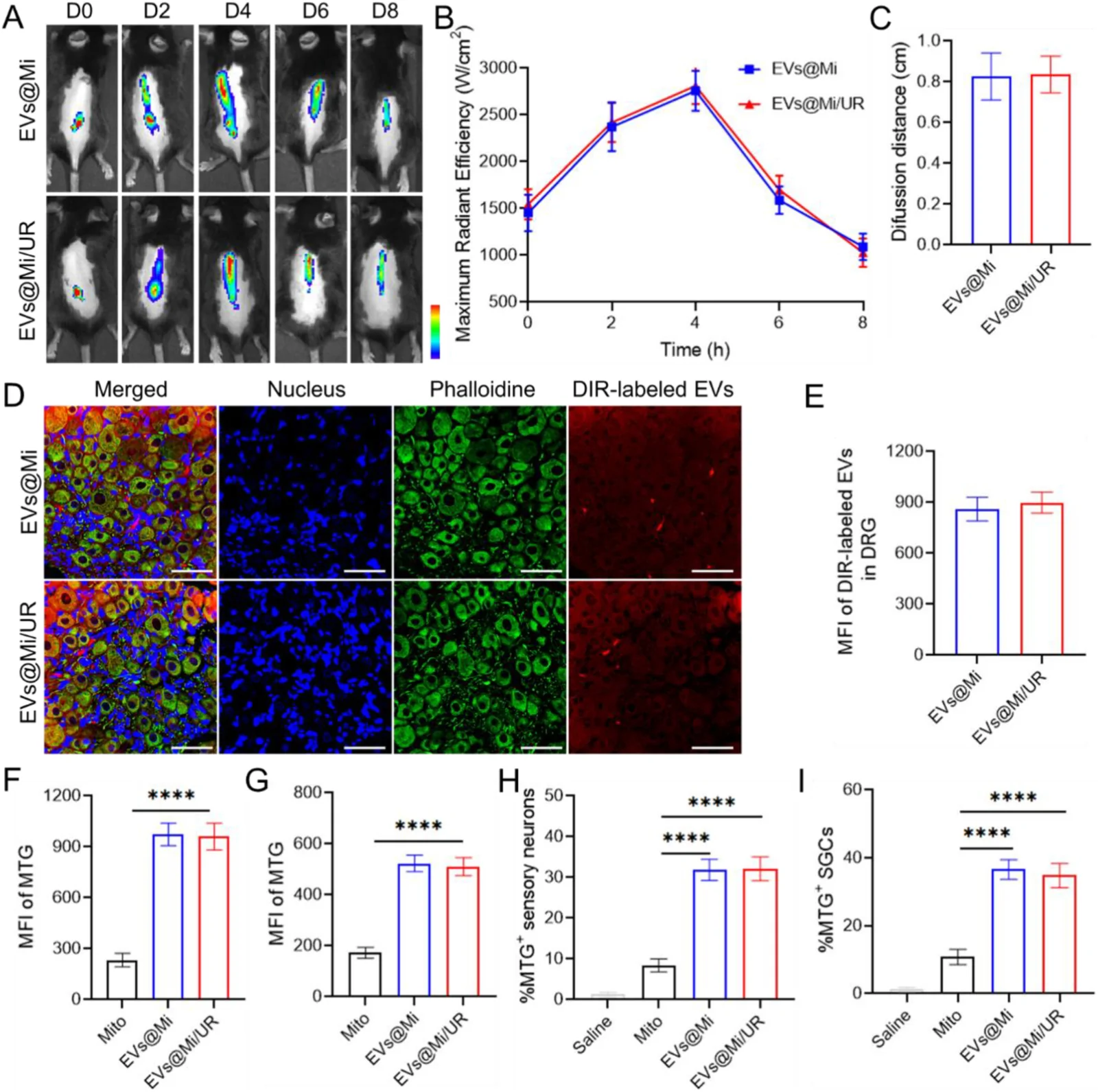
**Uptake of EVs@Mi/UR in vivo.** (A) Fluorescence imaging of mice after intrathecal injection with DiR-labeled EVs@Mi/UR and EVs@Mi. (B) The maximum radiant efficiency of intrathecal DiR-labeled EVs@Mi/UR and EVs@Mi at different time points, n = 3. (C) Maximum diffusion distance of EVs@Mi/UR and EVs@Mi from the injection site, n = 3. (D) The distribution of EVs@Mi/UR and EVs@Mi in DRG cells, n = 3. (E) Fluorescence quantitative analysis of the accumulation of EVs@Mi/UR and EVs@Mi in DRG, n = 3. (F-G) The MFI of MTG in isolated DRG sensory neurons (F) and SGCs (G) after treated with DiR-labeled EVs@Mi/UR and EVs@Mi for 4 days, n = 3. (H-I) MTG-positive DRG sensory neurons (H) and SGCs (I) isolated from mice after treated with DiR-labeled EVs@Mi/UR and EVs@Mi for 4 days, n = 3. Data are expressed as mean ± SD, *p < 0.05, **P < 0.01, ***p < 0.001, ****P < 0.0001.

For in vivo tracking, EVs@Mi/UR and EVs@Mi were labeled with DiR and administered intrathecally to mice. DiR-labeled EVs@Mi/UR and EVs@Mi distribute and diffuse along the spinal cord and were gradually cleared over 8 days post-injection (Fig. 4A). There were no obvious differences between EVs@Mi/UR and EVs@Mi in maximum radiant efficiency or spread distance (Fig. 4B and C). Subsequently, we explored the cellular internalization of EVs@Mi/UR and EVs@Mi with DRG cells. As shown in Fig. 4D and E, both EVs@Mi/UR and EVs@Mi were internalized by DRG cells, and fluorescence intensity in DRG were similar between two groups. To further validate the effective mitochondrial transplantation in vivo, the mitochondria in EVs@Mi/UR and EVs@Mi were labeled with MitoTracker Green FM (MTG, green). Subsequently, DRG sensory neurons and SGCs were isolated from mice 4 days after treated with EVs@Mi/UR and EVs@Mi. It could be found that MTG-labeled mitochondria were internalized by sensory neurons and SGCs. Additionally, the MFI of MTG and percentage of MTG-positive sensory neurons and SGCs in the EVs@Mi/UR and EVs@Mi treatment groups was similar and obviously higher than that in mice treated with free mitochondria (Fig. 4F–I). These results indicated the successful mitochondrial delivery by EVs@Mi/UR and EVs@Mi.

**Fig. 5:**
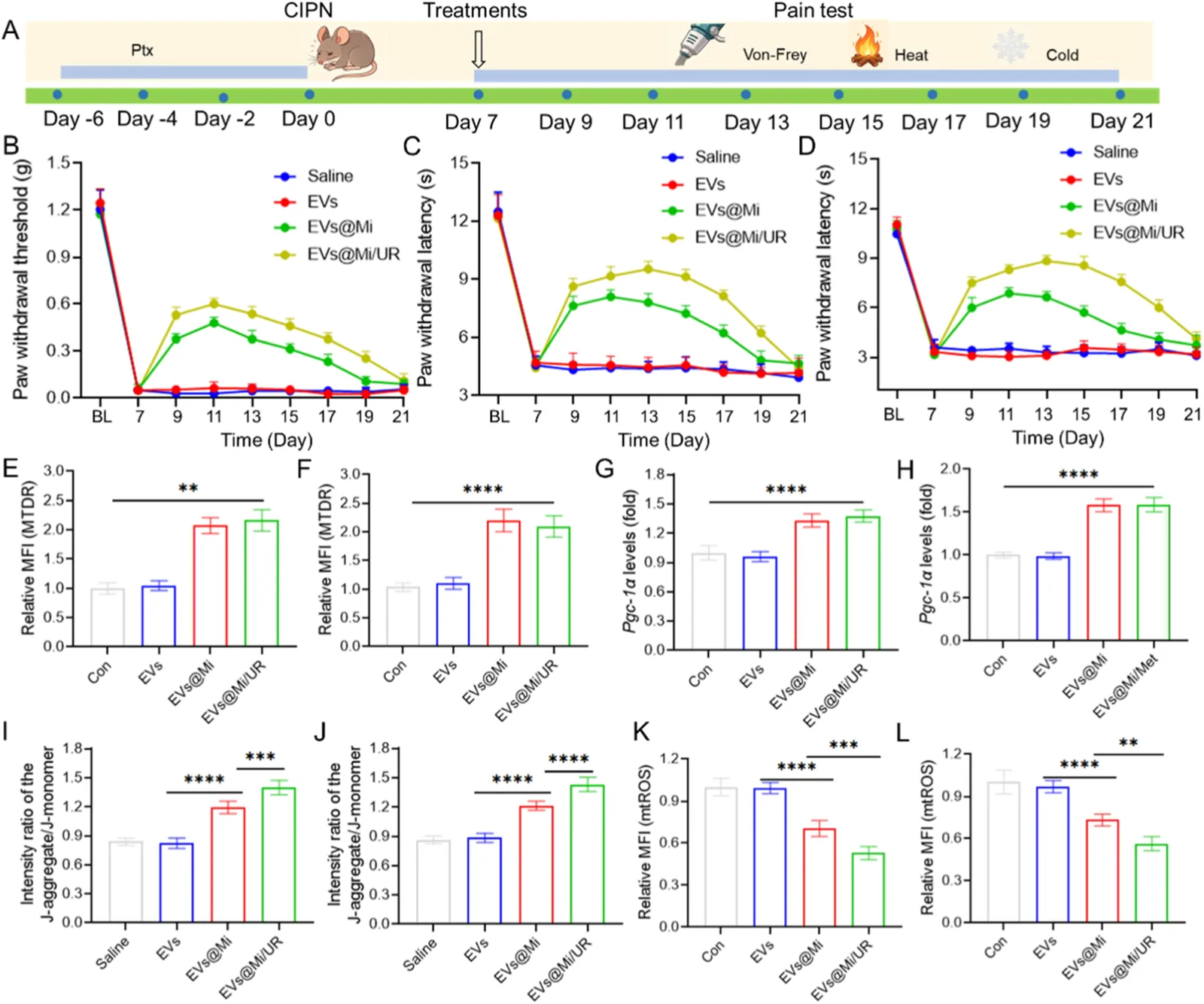
**In vivo relief of neuropathic pain in CIPN models.** (A) Protocol of treatments of EVs@Mi/UR in CIPN for behavioral test. (B) Mechanical allodynia examination following intrathecal administration of various formulations, n = 5. (C) Paw withdrawal latency of mice after various treatments in thermal hyperalgesia examination, n = 5. (D) Paw withdrawal latency of mice with of dry ice-induced cold stimulation, n = 5. (E-F) Relative MFI of MTDR in DRG sensory neurons (E) and SGCs (F) isolated from CIPN mice after various treatments, n = 5. (G-H) Levels of *Pgc-1*α in DRG sensory neurons (G) and SGCs (H), analyzed by qPCR. (I-J) The intensity ratio of the J-aggregate/J-monomer in DRG sensory neurons (I) and SGCs (J) after various treatments, n = 5. (K-L) Relative MFI of MitoSOX Red in DRG sensory neurons (K) and SGCs (L) after various treatments, n = 5. Data are expressed as mean = 5. (J)gregate/J-monom***p < 0.001, ****P < 0.0001.

Encouraged by their excellent mitochondrial delivery, we explored the ability of EVs@Mi/UR to relief neuropathic pain. CIPN lacks an identifiable pain source and can persist for months to years; current treatments are limited, with opioids offering only palliative benefit and risk of dependence. We investigated whether EVs@Mi/UR offered an alternative treatment to CIPN through mechanical allodynia and thermal/cold stimulation experiments (Fig. 5A). The von-Frey test indicated that EVs alone had no analgesic effect, whereas both EVs@Mi and EVs@Mi/UR reduced mechanical responses. EVs@Mi extended the duration of action to at least 10 days, while EVs@Mi/UR produced a greater intensity of relief (Fig. 5B). Similar patterns emerged in thermal hyperalgesia examination and dry ice-induced cold stimulation experiments (Fig. 5C and D). The withdrawal latency in response to the heat and cold stimulation was also significantly reduced following EVs@Mi/UR and EVs@Mi treatments. Compared with EVs@Mi treatment, EVs@Mi/UR treatment exhibited lower heat and cold sensitivity in the models. To elucidate the detailed mechanism underlying the enhanced pain relief of EVs@Mi/UR, we investigated the mitochondrial status in the DRG after various treatment. Consistent with the in vitro results, EVs@Mi/UR increased the mitochondrial mass and mtDNA copy numbers of DRG sensory neurons and SGCs isolated from DRGs of mice, (Fig. 5E–F and S15). It could be explained that EVs@Mi/UR improved the mitochondrial biogenesis, as indicated by the upregulation in levels of *Pgc-1α* and *Tfam* in both SGCs and sensory neurons (Fig. 5G–H and S16). Furthermore, EVs@Mi/UR led to a substantial increase in the ratio of J-aggregate/J-monomer intensities as well as significant decrease in mitoROS levels, which was better than EVs@Mi, suggesting the decrease in damaged mitochondria in DRG cells of CIPN mice (Fig. 5I–L). Furthermore, EVs@Mi/UR upregulated mitophagy-related genes in DRG sensory neurons and SGCs (Fig. S17). Collectively, these results indicated that EVs@Mi/UR could improve the mitochondrial status in DRGs for the relief of CIPN through simultaneously exogenous mitochondrial transplantation and endogenous mitochondrial renewal.

**Fig. 6:**
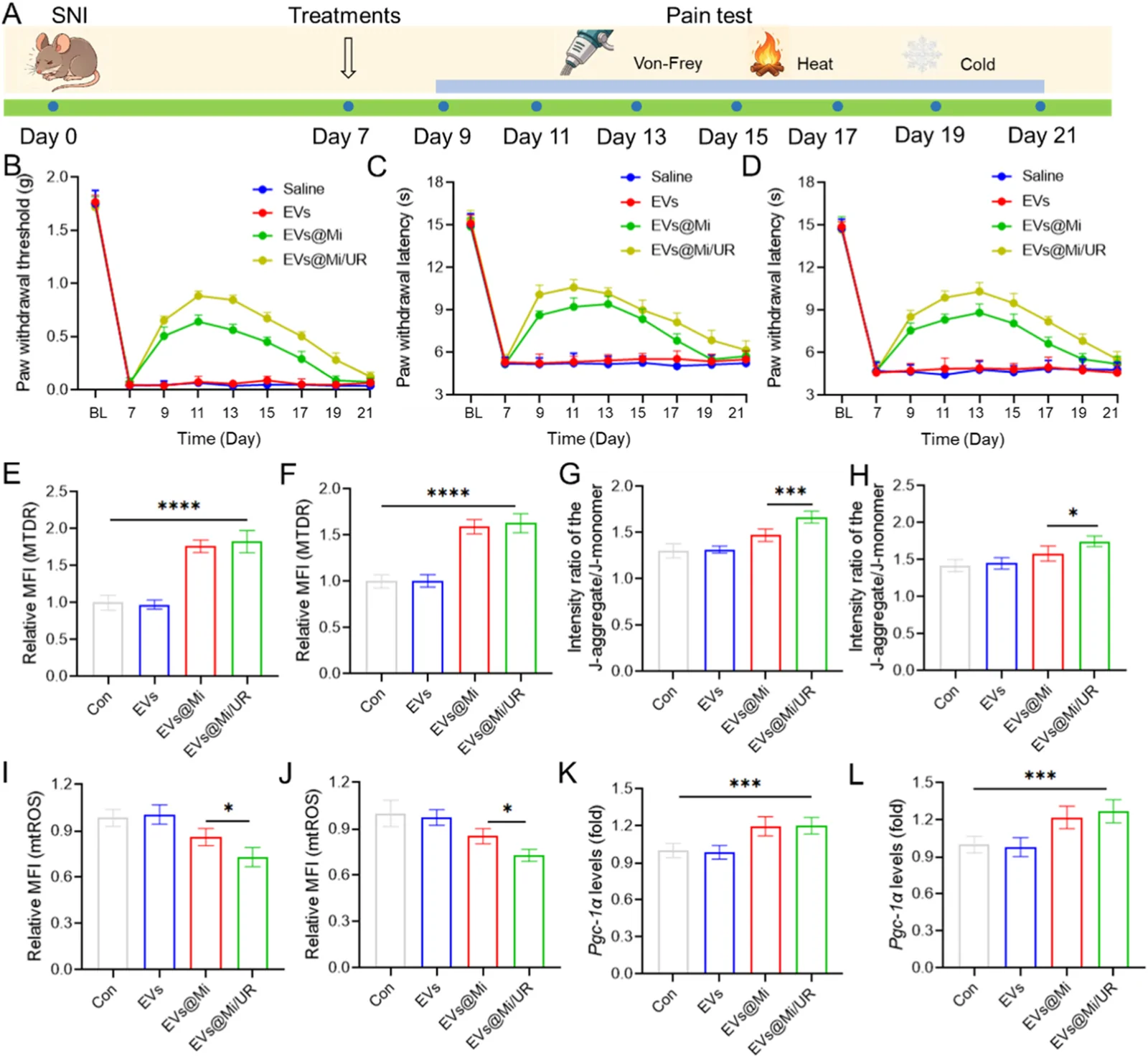
**In vivo relief of neuropathic pain in SNI models.** (A) Schematic timeline of the treatments of EVs@Mi/UR in SNI mice. (B) Mechanical allodynia assessment after intrathecal injection with EVs@Mi/UR in SNI mice, n = 5. (C) Paw withdrawal latency of mice after various treatments in thermal hyperalgesia test, n = 5. (D) Paw withdrawal latency of mice with of dry ice-induced cold stimulation, n = 5. (E-F) Relative MFI of MTDR in DRG sensory neurons (E) and SGCs (F) isolated from SNI mice after various treatments, n = 5. (G-H) The intensity ratio of the J-aggregate/J-monomer in DRG sensory neurons (G) and SGCs (H) isolated from SNI mice after various treatments, n = 5. (I-J) Relative MFI of MitoSOX Red in DRG sensory neurons (I) and SGCs (J) after various treatments, n = 5. (K-L) Levels of PGC-1ents, n = 5.RG sensory neurons (DRG sensory neurons (fromti Data are expressed as mean ± SD, *p < 0.05, **P < 0.01, ***p < 0.001, ****P < 0.0001.

The analgesic effect of EVs@Mi/UR was also investigated in SNI, an easy-to-operate model producing reproducible, robust, and long-lasting neuropathic pain through the ligation of the common peroneal and tibial nerves of the hind limb (Fig. 6A). Mechanical allodynia results revealed that the baseline (BL) of paw withdrawal threshold of mice prior to SNI modeling was around 2.0 g and severe mechanical allodynia was observed with the threshold sharply decreasing to approximately zero on the 7th day following SNI modeling, indicative of the success establishment of SNI models (Fig. 6B). Unlike CIPN, SGCs and sensory neurons isolated from SNI models didn't exhibit lower mitochondrial quality and mitochondrial quantity than those from healthy mice (Fig. S18). However, EVs@Mi and EVs@Mi/UR reduced the mechanical response and decreased noxious heat and cold sensitivity in the SNI models (Fig. 6B–D). Furthermore, EVs@Mi/UR showed a superiority to relieve the SNI pain compared with EVs@Mi. Notably, EVs@Mi/UR exhibited a better effect in improving the mitochondrial status in the DRG cells of SNI, as indicated by the elevated mitochondrial mass, increased ratio of the J-aggregate/J-monomer and decreased mtROS levels, which was better than EVs@Mi (Fig. 6G–J and S19). Furthermore, EVs@Mi/UR upregulated not only the mitochondrial biogenesis-related genes, but also the mitophagy-related genes, further confirming the combined effects of EVs@Mi/UR on exogenous mitochondrial transplantation and endogenous mitochondrial renewal (Fig. 6K–L and S20-21). These results demonstrate the ability of EVs@Mi/UR to alleviate SNI-induced pain, suggesting that mitochondrial intervention even in neurons without apparent mitochondrial damage can still exert a neuropathic pain-relieving effect.

Systemic adverse effects were evaluated in mice receiving repeated treatments. No significant weight loss occurred in EVs@Mi- or EVs@Mi/UR-treated groups compared to controls (Fig. S22). At study end, treatments with EVs@Mi and EVs@Mi/UR did not lead to the abnormalities of blood biochemistry index, including alkaline phosphatase (ALP), aspartate aminotransferase (AST), alanine aminotransferase (ALT) and total bilirubin (TBIL), blood urea nitrogen (BUN) and creatinine (CREA) serving as liver and kidney function indicators, as well as creatine kinase (CK) serving as a heart function indicator, as well as total cholesterol (TC) and triglyceride (TG) that reflected blood lipids (Fig. S23). Furthermore, H&E staining revealed no histopathological changes in heart, brain, liver, spleen, lung, or kidney across all groups (Fig. S24). These findings indicate low systemic toxicity of EVs@Mi/UR throughout treatment.

## 4 Conclusion

In this study, we demonstrated that DRG sensory neurons and SGCs in CIPN models exhibit profound deficits in mitochondrial quantity, quality, and biogenesis, along with impaired mitophagy. To address these issues, we engineered an EV-based nanoplatform (EVs@Mi/UR) that delivers functional mitochondria together with a mitophagy inducer, to simultaneously achieve exogenous mitochondrial supplementation and endogenous mitochondrial renewal in DRG cells. Compared with mitochondrial transplantation alone, this dual-modality strategy not only replenishes exogenous healthy mitochondria but also promotes the clearance of damaged endogenous mitochondria, leading to superior restoration of mitochondrial homeostasis and metabolic function. The platform achieved efficient DRG delivery after intrathecal injection and produced sustained analgesic effects in both CIPN and SNI models. Interestingly, even in SNI mice where baseline mitochondrial parameters appeared normal, EVs@Mi/UR still improved pain behaviors, suggesting that enhancing mitochondrial fitness can be beneficial beyond obvious dysfunction. Given its favorable biocompatibility and low systemic toxicity, EVs@Mi/UR represents a promising therapeutic candidate for neuropathic pain management. These findings highlight the broader therapeutic potential of mitochondrial remodeling for neuropathic pain of different etiologies.

We recognize several limitations in this study. In particular, we did not include a UA-only control (EVs@UR) or calculate a pharmacological synergy index. Thus, it is not yet possible to conclude whether the combined delivery of exogenous mitochondria and UA-induced mitophagy produces a true synergistic effect. Further studies incorporating dose-escalation designs and EVs@UR controls are warranted to systematically explore the potential crosstalk between these two therapeutic modalities.

## CRediT authorship contribution statement

**Xianquan An:** Writing – original draft, Methodology, Investigation, Conceptualization. **Li Zhang:** Methodology, Investigation, Data curation. **Xin Zhang:** Methodology, Investigation, Formal analysis. **Meidan Fang:** Software, Methodology, Formal analysis. **Cong Wang:** Software, Investigation, Data curation. **Yu Han:** Visualization, Formal analysis. **Zheng Wang:** Visualization, Methodology, Investigation. **Jinfeng Wang:** Writing – review & editing, Supervision, Project administration. **Shuting Zuo:** Writing – review & editing, Supervision, Project administration, Investigation, Conceptualization.

## Ethics and consent to participate declarations

Not applicable.

## Funding declaration

This work was supported by the 10.13039/100007847Jilin Provincial Natural Science Foundation (YDZJ202401188ZYTS), the Natural Science Foundation of Xuzhou Medical University (D2025008) and the Chronic Disease Management Research Project of 10.13039/501100018684National Health Commission Capacity Building and Continuing Education Center (GWJJMB202510042029).

## Declaration of competing interests

The authors declare that they have no known competing financial interests or personal relationships that could have appeared to influence the work reported in this paper.

## Data Availability

Data will be made available on request.
