# Supplementary material for: Mitochondrial remodeling by extracellular vesicles co-delivering functional mitochondria and a mitophagy inducer for neuropathic pain relief
Source: Mater Today Bio. 2026 Sep 1;40:103636. doi: 10.1016/j.mtbio.2026.103636 (PMC13571700; doi:10.1016/j.mtbio.2026.103636)
Supplement: Supplementary file 1 [file mmc1.docx]

Supporting Information

**Mitochondrial Remodeling by Extracellular Vesicles Co‑Delivering Functional Mitochondria and a Mitophagy Inducer for Neuropathic Pain Relief**

Xianquan An^1^, Li Zhang^1^, Xin Zhang^1^, Meidan Fang^1^, Cong Wang^1^, Yu Han^1^, Zheng Wang^4, 5^, Jinfeng Wang^3,^ *, Shuting Zuo^2,^ *

^1^ Department of Anesthesiology, The Second Hospital of Jilin University, Changchun 130041, China

^2^ Department of Breast Surgery, The Second Hospital of Jilin University, Changchun 130041, China

^3^ Department of Anesthesiology and Pain Medicine，Suzhou TCM Hospital Affiliated to Nanjing University of Chinese Medicine, Suzhou 215009, China

^4^ CAS Key Laboratory of Nano-Bio Interface, Division of Nanobiomedicine, Suzhou Institute of Nano-Tech and Nano-Bionics, Chinese Academy of Sciences, Suzhou 215123, China

^5^ Jiangsu Key Laboratory of New Drug Research and Clinical Pharmacy, School of Pharmacy, Xuzhou Medical University, Xuzhou 221004, China

**Figure S1.** **Mitochondrial mass of SGCs.** Relative mean fluorescence intensity (MFI) of MTDR in SGCs of CIPN model mice, n = 3. Data are expressed as mean ± SD, n = 3, *p<0.05, **P<0.01, ***p<0.001, ****P<0.0001.

**Figure S2.** **Mitochondrial contents of DRG cells.** Relative counts of mitochondrial DNA (mtDNA) copies in sensory neurons (A) and SGCs (B) from CIPN mice, n = 3. Data are expressed as mean ± SD, n = 3, *p<0.05, **P<0.01, ***p<0.001, ****P<0.0001.

**Figure S3.** **Levels of mtROS in SGCs.** Relative MFI of MitoSOX Red in SGCs of CIPN model mice, n = 3. Data are expressed as mean ± SD, n = 3, *p<0.05, **P<0.01, ***p<0.001, ****P<0.0001.

**Figure S4. Mitochondrial membrane potential assays of SGCs.** (A) Confocal images of mitochondrial membrane potential in SGCs of healthy and CIPN mice, the scale bar = 20 μm. (B) Flow cytometry analysis of the intensity ratio of the J-aggregate/J-monomer in SGCs of healthy and CIPN mice, n = 3. Data are expressed as mean ± SD, n = 3, *p<0.05, **P<0.01, ***p<0.001, ****P<0.0001.

**Figure S5. Membrane potential analysis of DRG in CIPN models.** Flow cytometry assays of the relative MFI of TMRM in sensory neurons (A) and SGCs (B) from CIPN mice, n = 3. Data are expressed as mean ± SD, n = 3, *p<0.05, **P<0.01, ***p<0.001, ****P<0.0001.

**Figure S6.** Quantitative analysis of the non-mitochondrial respiration and proton leak (A) as well as coupling efficiency (B)of DRG sensory neurons from control and CIPN mice according to the OCR, n = 3. Data are expressed as mean ± SD, *p<0.05, **P<0.01, ***p<0.001, ****P<0.0001.

**Figure S7.** Quantitative qPCR analysis of mitochondrial biogenesis genes including *Pgc-1β* (A, B), and *Prkn* (C, D) in the DRG sensory neurons (A, C) and SGCs (B, D). Data are expressed as mean ± SD, *p<0.05, **P<0.01, ***p<0.001, ****P<0.0001.

**Figure S8. Cytotoxicity of EVs@Mi/UR.** Cellular viability of sensory neurons (A) and SGCs (B) after incubated with various concentration of EVs@Mi and EVs@Mi/UR for 24 h, n = 5. Data are presented as the mean ± SD.

**Figure S9. Cellular internalization with SGCs.** Quantitative analysis of the cellular internalization of EVs@Mi, EVs@Mi/UR and free mitochondria with SGCs after 3h of incubation, n = 3. Data are expressed as mean ± SD, *p<0.05, **P<0.01, ***p<0.001, ****P<0.0001.

**Figure S10. Mitochondrial content of SGCs after various treatments.** (A) Relative MFI of MTDR in SGCs after various treatments, n = 3. (B) The number of mtDNA copies in SGCs, n = 3. Data are expressed as mean ± SD, *p<0.05, **P<0.01, ***p<0.001, ****P<0.0001.

**Figure S11. Mitochondrial damage of SGCs after various treatments.** (A) Relative MFI of TMRM in SGCs after various treatments, n = 3. (B) Flow cytometry analysis of the intensity ratio of the J-aggregate/J-monomer in SGCs after various treatments, n = 3. (C) Relative MFI of MitoSOX Red in SGCs after various treatments. Data are expressed as mean ± SD, *p<0.05, **P<0.01, ***p<0.001, ****P<0.0001.

**Figure S12.** Quantitative qPCR analysis of *Pgc-1β* levels in the DRG sensory neurons (A) and SGCs (B). Data are expressed as mean ± SD, *p<0.05, **P<0.01, ***p<0.001, ****P<0.0001.

**Figure S13.** Quantitative analysis of basal, maximal, and spare respiratory capacity and ATP production rate of DRG sensory neurons after various treatments, according to the OCR. Data are expressed as mean ± SD, n = 3.

**Figure S14.** Quantitative analysis of the non-mitochondrial respiration and proton leak (A) as well as coupling efficiency (B)of DRG sensory neurons after various treatments, according to the OCR., n = 3. Data are expressed as mean ± SD, *p<0.05, **P<0.01, ***p<0.001, ****P<0.0001.

**Figure S15. Mitochondrial contents in DRG cells of CIPN mice.** Relative counts of mtDNA copies in sensory neurons (A) and SGCs (B) isolated from CIPN mice after various treatments, n = 5. Data are expressed as mean ± SD, *p<0.05, **P<0.01, ***p<0.001, ****P<0.0001.

**Figure S16. Mitochondrial biogenesis-related genes in DRG cells of CIPN mice.** Levels of *Tfam* in sensory neurons (A) and SGCs (B) isolated from CIPN mice after various treatments, analyzed by qPCR, n = 5. Data are expressed as mean ± SD, *p<0.05, **P<0.01, ***p<0.001, ****P<0.0001.

**Figure S17. Mitophagy-related genes in DRG cells of CIPN mice.** Levels of *Pink1* in sensory neurons (A) and SGCs (B) isolated from CIPN mice after various treatments, analyzed by qPCR, n = 5. Data are expressed as mean ± SD, *p<0.05, **P<0.01, ***p<0.001, ****P<0.0001.

**Figure S18. Mitochondrial status in DRG cells of SNI mice.** (A-B) Relative MFI of MTDR in DRG sensory neurons (B) and SGCs (B) isolated from SNI mice, n = 5. (C-D) Relative counts of mitochondrial DNA (mtDNA) copies in sensory neurons (C) and SGCs (D) from SNI mice, n = 5. (E-F) Relative MFI of MitoSOX Red in sensory neurons (E) and SGCs (F) from SNI mice, n = 5. (G-H) Flow cytometry analysis of the intensity ratio of the J-aggregate/J-monomer in sensory neurons (G) and SGCs (H) isolated from SNI mice, n = 5. Data are expressed as mean ± SD.

**Figure S19. Mitochondrial contents in DRG cells of SNI mice.** Relative counts of mtDNA copies in sensory neurons (A) and SGCs (B) isolated from SNI mice after various treatments, n = 5. Data are expressed as mean ± SD, *p<0.05, **P<0.01, ***p<0.001, ****P<0.0001.

**Figure S20. Mitochondrial biogenesis-related genes in SNI cells of CIPN mice.** Levels of *Tfam* in sensory neurons (A) and SGCs (B) isolated from SNI mice after various treatments, analyzed by qPCR, n = 5. Data are expressed as mean ± SD, *p<0.05, **P<0.01, ***p<0.001, ****P<0.0001.

**Figure S21. Mitophagy-related genes in DRG cells of SNI mice.** Levels of *Pink1* in sensory neurons (A) and SGCs (B) isolated from CIPN mice after various treatments, analyzed by qPCR, n = 5. Data are expressed as mean ± SD, *p<0.05, **P<0.01, ***p<0.001, ****P<0.0001.

**Figure S22.** Body weights of the mice in each group, n =5. Data were presented as mean ± SD.

**Figure S23.** **Blood biochemistry index of the mice after various treatments**. (A) alkaline phosphatase (ALP), (B) aspartate aminotransferase (AST), (C) alanine aminotransferase (ALT), (D)blood urea nitrogen (BUN), (E) Creatine Kinase (CK), (F)creatinine (CREA), (G) total bilirubin (TBIL), (H) total cholesterol (TC) and (G) triglyceride (TG) levels. Data were presented as mean ± SD, n = 5. Data are expressed as mean ± SD.

**Figure S24.** H&E staining images of main organs of the mice after treated with saline, EVs@Mi, and EVs@Mi/UR. The scale bar represents 100 μm.
